# Association study of *WNK1* genetic variants and essential hypertension risk in the Northern Han Chinese in Beijing

**DOI:** 10.3389/fgene.2023.1234536

**Published:** 2023-09-15

**Authors:** Kuo Liu, Jielin Liu, Ya Liu, Hao Wang, Zuoguang Wang, Jinghua Liu, Shaojun Wen

**Affiliations:** ^1^ Department of Hypertension Research, Beijing Anzhen Hospital, Beijing Institute of Heart Lung and Blood Vessel Diseases, Capital Medical University, Beijing, China; ^2^ Department of Cardiology, Luoyang Central Hospital Affiliated to Zhengzhou University, Luoyang, Henan, China; ^3^ Department of Cardiology, Beijing Anzhen Hospital, Beijing Institute of Heart Lung and Blood Vessel Diseases, Capital Medical University, Beijing, China

**Keywords:** lysine deficient protein kinase 1, essential hypertension, genetic variant, Northern Han Chinese population, case-control study

## Abstract

**Background:** Essential hypertension (EH) is a complex disorder resulting from interaction of genetic and environmental factors. Lysine deficient protein kinase 1 (WNK1) plays a very important role in maintaining renal potassium, sodium and chlorine ions balance as well as the regulation of blood pressure, so the *WNK1* gene is considered a key gene for EH. This study thus sought to evaluate possible genetic associations between the *WNK1* genetic variants and EH risk in the Northern Han Chinese population in Beijing.

**Methods:** This study included 476 hypertensive subjects and 491 normotensive subjects. A total of 12 tag SNVs of *WNK1* gene were genotyped successfully by TaqMan assay. Comparisons of the genotypic and allelic frequency between cases and controls were made by using the chi-square test. Logistic regression analyses were performed under different genetic models, and haplotype analysis was also conducted.

**Results:** A total of 12 SNVs were identified as the tag SNVs for *WNK1* gene. Significant associations were observed between *WNK1* gene rs7305099 variant and EH risk, and T allele influenced hypertension risk in a protective manner. After correcting for multiple testing using Bonferroni, the significance remained for the SNV of rs7305099 in three genetic models [allele comparison, *p* < 0.0002, OR = 0.627, 95%CI (0.491–0.801); homozygote comparison, *p* < 0.0003, OR = 0.278, 95%CI (0.140–0.552); additive model, *p* < 0.0003, OR = 0.279, 95%CI (0.140–0.553)]. In the haplotype analyses, we found that the haplotype A-A-A-C-G-G-G was significantly associated with increased risk for EH (*p* = 0.043, OR = 1.23).

**Conclusion:** Our data suggested that the rs7305099 genetic variant and the haplotype A-A-A-C-G-G-G on *WNK1* gene might be associated with the susceptibility of EH in the Northern Han Chinese population. These could provide evidences to the risk assessment, early prevention and individualized therapy of EH to some extent.

## 1 Introduction

Essential hypertension (EH) is a major global health concern that contributes to millions of disability and premature death worldwide every year due to cardio-cerebrovascular events, congestive heart failure, and endstage renal disease (Mills et al., 2016). The recent survey published on Circulation showed that approximately 23.2% (≈244.5 million) of the Chinese adult population ≥18 years of age had hypertension During 2012 and 2015 (Wang et al., 2018). Hypertension in Northern China is especially a serious problem, with high prevalence and a lack of control (Xu et al., 2021). The etiology and pathogenesis of EH are regarded as a highly heterogeneous disorder resulted from multiple genetic and environmental factors, as well as their complex interactions. Evidences from pedigree study have demonstrated that genetic factors are a determinant of hypertension to a large extent (Hong et al., 1994). Approximately 20%–60% of the blood pressure (BP) variation among the general population is genetically determined (Levy et al., 2000). Therefore, identification for the potential genes susceptible to hypertension is of crucial importance, which would help to realize the early detection of individuals, treatment and prevention of hypertension.

WNK1 (lysine deficient protein kinase 1) is a member of the WNKs subfamily, the gene of which was identified a candidate gene for BP regulation and EH (Lifton et al., 2001; Wilson et al., 2001; Zambrowicz et al., 2003). Human *WNK1* maps to chromosome 12p13.33, is encoded by 31 exons, and spans over 160 kb of genomic DNA. There are two major isoforms of WNK1, a kinase-active full long isoform (L-WNK1) with complete kinase domain and a kinase-deficient kidney specific short isoform (KS-WNK1). L-WNK1 is ubiquitously expressed in various tissues, particularly in heart and muscle, whereas KS-WNK1 is produced mainly in kidney (Delaloy et al., 2003). It is thought that the elevation of BP in PHA2 patients may partially due to increasing reabsorption of water and sodium via L-WNK1/KS-WNK1 mediated upregulation of Na-Cl cotransporter (NCC), resulting volume expansion-induced hypertension (Vidal-Petiot et al., 2013; Brown et al., 2021; Meor Azlan et al., 2021). Hence, *WNK1* is an obvious candidate gene, and the possibility has been proposed that genetic variants in *WNK1* alter susceptibility to EH.

Highly polymorphic was observed in *WNK1*, with >100 validated single nucleotide variant (SNVs). The SNV rs1468326, located 3 kb from the *WNK1* promoter, was first found to be associated with severity of hypertension in families from the British Genetics of Hypertension (BRIGHT) Study in 2005 (Newhouse et al., 2005). Subsequently, a case-control study in the Chinese Han population suggested allele A carriers of rs1468326 in *WNK1* had higher risk for EH by Han et al. (2011). In accordance with the Chinese study, Persu et al. (2016) reported the rs1468326 was associated with hypertension in multicenter study of white European populations. Additionally, consistent result was found that the SNV rs1468326 associated with EH for Tibetan individuals in China, which supports the role of *WNK1* as potential hypertension susceptibility genes (Shi et al., 2018). However, among the coding regions of *WNK1*, conflicting results were reported regarding the association of common variants and EH. Kokubo et al. (2004) first tested for the association among 7 *WNK1* SNVs (rs956868, rs3858703, and rs2286007 et al.) and EH in Japanese population of 771 hypertensives and 1047 controls selected from within the Suita cohort, but no association was found. However, Han and Hui (2008) reported the rs956868, located in the *WNK1* exon 13, was significantly associated with EH in the Han Chinese population (dominant model, AA + AC vs. CC: *p* = 0.007, OR = 1.39, 95%CI [1.10–1.77]) after adjusting the covariates by logistic regression analysis. Moreover, significant association was observed between the *WNK1* exon 1 variant (rs34880640) and EH in the Hani minority of China, but no positive result was found in the Yi minority (Cun et al., 2011). In aspect of *WNK1* introns, Newhouse et al. (2009) reported the major allele (A) of rs765250, located in intron 1, demonstrated the evidence for association with hypertension (*p* = 0.01, OR = 1.3, 95%CI [1.0–1.7]), whereas others failed to replicate this finding (Persu et al., 2016; Shi et al., 2018). Another SNV, rs880054 is in the 10th intron of *WNK1* gene. Tobin et al. (2005) found statistically significant association of rs880054 in *WNK1* with BP variation. Nevertheless, another study in the Tibetan population of China showed no significant association between rs880054 variant with hypertension (Shi et al., 2018). Therefore, the relationship of *WNK1* SNVs with EH was still a worthy research problem, and further studies are in needed. In the present study, we investigated the association between the SNV loci of *WNK1* gene and EH risk in Northern Han Chinese in the Beijing area.

## 2 Materials and methods

### 2.1 Ethics statement

The study complies with the Declaration of Helsinki. The aim, design, and detailed protocol of this study were approved by the local ethics committee of Beijing Anzhen Hospital of the Capital University of Medical Sciences. Written informed consent was obtained from each participant before enrollment.

### 2.2 Study population

Unrelated subjects were recruited to participate in this case-control study. All individuals selected for the study were of Northern Han Chinese origin in Beijing and did not migrate within three generations. Participants of Anzhen community were recruited from the physical examination center of Anzhen Hospital affiliated to Capital Medical University, Beijing, China, and another two examination centers at local health stations, Liuliqiao and Guozhuang, in Beijing suburbs. A standard questionnaire on personal medical history and family history of hypertension were completed for all subjects. All the participants were asked to avoid alcohol, cigarettes, coffee, tea and exercise for at least 1 h before the measurements. The BP measurements were accurately performed with a standard mercury sphygmomanometer by well-trained physicians according to a common protocol adapted from procedures recommended by European Society of Hypertension (Mancia et al., 2013). Measurements were taken at the right arm using the appropriate bladders after the participants had been seated on a chair with their feet on the floor and their arms supported at heart level for 10 min.

Hypertension was defined as the average systolic blood pressure (SBP) ≥140 mmHg and/or the average diastolic blood pressure (DBP) ≥90 mmHg and/or self-reported current treatment for hypertension with antihypertensive medication. The control subjects had SBP <140 mmHg and DBP <90 mmHg, respectively, and should never been diagnosed as hypertension or treated for hypertension (Mancia et al., 2013). After sitting for 30 min in a quiet room, three consecutive BP measurements were obtained at a 5-min interval, and the average BP was recorded. Subjects with secondary hypertension, white coat hypertension, diabetes mellitus, hepatic and renal dysfunction, serious cardiovascular and cerebrovascular diseases, malignant tumor, multiple organ failure or other serious diseases were excluded. In addition, pregnant or lactating women, gestational hypertension and pregnancy with hypertension were also excluded in our study. A questionnaire, physical examination, blood samples were administered to each of the participants. Information on smoking and drinking habits were obtained by interview. Smokers were defined as cigarette consumers who had smoked ≥100 cigarettes; and drinkers were defined as alcohol consumers who had drank ≥12 times during the past year (Ge et al., 2005; Gu et al., 2006).

### 2.3 SNV identification

The tag SNV approach was used to predict the common SNVs in the current study. The *WNK1* common SNVs were searched from the Han Chinese data sets of the International HapMap Project SNV database (http://www.hapmap.org/, HapMap Genome Browser release #27). Quality control (QC) filters were then applied to the SNVs and samples before analysis to ensure robust association tests. The following SNVs were excluded: those with a missing call rate of more than 15% or a minor allele frequency (MAF) of less than 10%. Then, the sets tag SNVs were selected to predict the remaining common SNVs with an *r* ^2^ ≥ 0.85 examined by Haploview 4.2 software (http://www.broad.mit.edu/mpg/haploview).

### 2.4 DNA preparation and genotyping

A 5-mL peripheral venous blood sample was collected for all participants after 12-h overnight fasting and taken into ethylene-diamine tetra-acetic (EDTA)-containing tubes. Genomic DNA was extracted from peripheral blood leukocytes with a standard phenol-chloroform method, and stored at −80°C for batch genotyping. All selected SNVs were genotyped using the TaqMan assay according to the manufacturer’s standard protocols. The *WNK1* SNV Taqman probes and primers, which were labeled with different fluorescent dyes, were obtained from Applied Biosystems Assay-by-Design Service for SNV genotyping (Applied Biosystems, Foster City, CA, United States). The sample DNA was amplified by PCR following the recommendations of the manufacturer. Genotyping reactions contained 1×TaqMan^®^ PCR Master Mix, No AmpErase^®^ UNG, and approximately 5 ng of genomic DNA in a final volume of 5 μL. Thermal cycling was done on the GeneAmp PCR System 9700 thermal cycler (Applied Biosystems). The cycling conditions were as follows: initial denaturation and activation at 95°C for 10 min, followed by 35 cycles of 95°C for 20 s, and 62°C for 1 min. Genotypes were differentiated by analyzing the fluorescence levels of PCR products using the ABI PRISM 7900HT Sequence Detector (Applied Biosystems). Moreover, genotyping was performed blindly to all other data.

### 2.5 Sample power calculation

Statistical power calculation was performed by using Quanto software (version 1.2.4, University of Southern California), and of at least 80% was considered adequate (Gauderman, 2002). The statistical power was prospectively estimated with the following parameters: an unmatched case-control study, a dominant genetic model, a prevalence of 23.2% of EH in China, an expected OR value of 1.5 (Wacholder et al., 2004). Statistical significance was set at *p* < 0.05. Both the prospective and *post hoc* statistical power were calculated.

### 2.6 Statistical analysis

Normally continuous variables were shown as mean ± standard deviation (SD), and categorical variables were expressed as frequencies. All the database management and statistical analyses were performed using the SPSS statistical software package (version 22.0; IBM SPSS Statistics). Comparisons between groups were made with Student’s *t*-test, Mann-Whitney *U* test and chi-squared test for normally distributed continuous, non-normally distributed continuous and categorical variables, respectively. The presence of Hardy–Weinberg equilibrium (HWE) was assessed by the chi-square test for goodness of fit based on a web program (http://ihg.gsf.de/cgi-bin/hw/hwa1.pl). The genotypic and allelic frequency between hypertensive group and control group were compared by using the chi-square test. Binominal logistic regression was used to examine the association between *WNK1* SNVs and the risk for hypertension under different genetic models (allele, dominant, recessive, homozygote, and additive comparison) after adjusting for potential confounding factors. Odds ratio (ORs) and their 95% confidence interval (95% CI) were calculated, while Bonferroni correction was applied for multiple comparisons based on the number of individual SNVs and genetic models examined (0.05/12/5). Thus, a corrected *p*-value threshold of 0.0008 was used to determine the Bonferroni-corrected statistical significance. Construction of the linkage disequilibrium (LD) map and haplotype blocks within *WNK1* SNVs were conducted based on genotypes using Haploview software (version 4.2) (http://www.broad.mit.edu/mpg/haploview/). Considering the effect of the covariates on the association analysis, the haplotype-based logistic regression analysis was performed using the PLINK software (Version 2.0) (http://pngu.mgh.harvard.edu/∼purcell/plink/). All haplotypes with frequency greater than 1% in our study were investigated. Haplotype-specific (HS) testing was also conducted to compare a specific haplotype with the others. The comparison method is a target haplotype compared with all remaining haplotypes merged. All statistical tests were two-tailed, and *p* < 0.05 was established statistically significant.

## 3 Results

### 3.1 Characteristics of the participants

According to the inclusion criteria, we initially included a total of 1120 subjects, in which 153 subjects were excluded based on exclusion criteria. The flow chart summarizing the process of subjects selection and reasons for exclusion was presented in the Supplement. Altogether 967 unrelated participated subjects comprising 476 hypertensive cases (275 males and 201 females; mean age 53.94 ± 9.49 years) and 491 normotensive controls (274 males and 217 females; mean age 54.21 ± 8.55 years) were finally recruited for the present study. The basic clinical characteristics and laboratory parameters of EH and NT groups were listed in Table 1. Age and gender were adequately matched between the two groups. In both total and gender stratified subgroups, there were no significant differences between hypertensive patients and healthy normotensive subjects in gender, age, heart rate (HR), and creatinine (Cr), as well as incidences of smokers and drinkers. The patients with hypertension had relatively higher body mass index (BMI), systolic blood pressure (SBP), diastolic blood pressure (DBP), glucose (Glu), triglyceride (TG), and uric acid (UA) levels, compared with the control group, in both total and male subgroup. However, levels of total cholesterol (TC), high-density lipoprotein cholesterol (HDL-C), and low-density lipoprotein cholesterol (LDL-C) were found to be lower in hypertension group. In addition, the similar trend was also observed in the subgroup of female. The levels of BMI, SBP, DBP, Glu, TG, and Hcy were relatively higher in hypertension group. The plasma concentration of the HDL-C was found to be lower in the female hypertensive group as compared to the female normotensive group. Moreover, a medication survey regarding the hypertensive patients in our study showed that 78.57% of individuals taked antihypertensive drugs regularly according to medical advice, while 21.43% of individuals did not take medication or take antihypertensive drugs irregularly.

**TABLE 1:** Basic clinical characteristics of the participants.

	Overall			Male			Female		
	Hypertension (*n* = 476)	Control (*n* = 491)	*p*	Hypertension	Control	*p*	Hypertension	Control	*p*
Gender (Male/Female)	275/201	274/217	0.537	275	274	-	201	217	-
Age (years)	53.94 ± 9.49	54.21 ± 8.55	0.464	52.51 ± 9.20	53.13 ± 8.39	0.214	55.91 ± 9.56	55.58 ± 8.57	0.571
BMI (kg/m^2^)	26.29 ± 3.48	24.59 ± 3.07	<0.001^b^	27.20 ± 3.25	25.33 ± 3.03	<0.001^b^	24.94 ± 3.39	23.61 ± 2.86	<0.001^b^
HR (bpm)	69.04 ± 9.73	69.17 ± 8.93	0.515	67.96 ± 9.04	68.65 ± 9.40	0.391	70.50 ± 10.44	69.84 ± 8.25	0.482
SBP (mmHg)^a^	136.18 ± 17.61	115.39 ± 10.86	<0.001^b^	135.15 ± 16.42	117.00 ± 9.84	<0.001^b^	137.57 ± 19.06	113.38 ± 11.74	<0.001^b^
DBP (mmHg)^a^	85.96 ± 11.51	73.14 ± 8.62	<0.001^b^	87.55 ± 10.78	75.89 ± 7.54	<0.001^b^	83.80 ± 12.13	69.67 ± 8.65	<0.001^b^
GLU (mmol/L)	5.40 ± 0.72	5.23 ± 0.71	<0.001^b^	5.43 ± 0.79	5.29 ± 0.57	0.027^b^	5.36 ± 0.62	5.16 ± 0.85	<0.001^b^
Cr (μmol/L)	72.81 ± 16.59	71.41 ± 13.95	0.511	81.03 ± 15.33	80.27 ± 10.50	0.682	61.50 ± 10.49	60.21 ± 8.73	0.245
TC (mmol/L)	4.94 ± 1.00	5.16 ± 0.92	<0.001^b^	4.91 ± 1.02	5.18 ± 0.93	0.001^b^	4.99 ± 0.97	5.15 ± 0.90	0.078
TG (mmol/L)	1.91 ± 1.59	1.54 ± 0.94	<0.001^b^	2.04 ± 1.76	1.71 ± 1.02	0.023^b^	1.73 ± 1.30	1.33 ± 0.79	<0.001^b^
HDL-C (mmol/L)	1.17 ± 0.60	1.26 ± 0.36	<0.001^b^	1.09 ± 0.26	1.17 ± 0.39	0.005^b^	1.28 ± 0.86	1.36 ± 0.30	<0.001^b^
LDL-C (mmol/L)	3.00 ± 0.87	3.21 ± 0.84	<0.001^b^	3.00 ± 0.89	3.30 ± 0.84	<0.001^b^	3.00 ± 0.86	3.1 ± 0.83	0.252
UA (μmol/L)	337.17 ± 100.22	315.40 ± 79.96	<0.001^b^	380.45 ± 97.79	354.59 ± 71.37	<0.001^b^	277.79 ± 68.05	265.65 ± 60.25	0.055
Hcy (μmol/L)	13.26 ± 6.16	12.81 ± 7.93	0.057	15.14 ± 7.10	14.66 ± 7.83	0.376	10.74 ± 3.19	10.45 ± 7.45	0.013^b^
Smokers (n, %)	171 (51.2)	163 (48.8)	0.373	161 (50.9)	155 (49.1)	0.639	10 (55.6)	8 (44.4)	0.517
Drinkers (n, %)	208 (49.2)	215 (50.8)	0.977	188 (50.3)	186 (49.7)	0.854	20 (40.8)	29 (59.2)	0.287

Continuous variables were expressed as means ± standard deviations when normally distributed and as median (interquartile range) when asymmetrically distributed. Abbreviations: BMI, body mass index; Cr, creatinine; DBP, diastolic blood pressure; Glu, glucose; Hcy, homocysteine; HDL-C, high-density lipoprotein cholesterol; HR, heart rate; LDL-C, low-density lipoprotein cholesterol; SBP, systolic blood pressure; TC, total cholesterol; TG, triglyceride; UA, uric acid.

a The well-treated patients were also included in Hypertension group.

b *p* < .05.

### 3.2 Detection and distribution of the SNVs

In this study, 14 tag SNVs (rs11064524, rs4980974, rs11608756, rs7305099, rs880054, rs12828016, rs2051852, rs4980973, rs10849558, rs10774461, rs11611231, rs956868, rs7972490, and rs10774457) of the *WNK1* gene were selected. The genotyping success rates (GSRs) for the 14 SNVs were all over 90%, and the MAFs of the SNVs were all more than 0.10. Two SNVs (rs10849558 and rs10774457) in *WNK1* were deviated significantly from HWE (all *p* < 0.05) for healthy controls, and were excluded from the further analysis. Table 1 in the Supplement shows the distributions of genotype and allele frequencies in total and gender subgroups, as well as HWE tests for 12 SNVs. Chi-square analyses showed that the genotype distributions of four SNVs (rs7305099, rs880054, rs12828016, and rs7972490) were significantly different (*p* < 0.05) between EH and NT groups. In the subgroup by gender, significant differences for four SNVs (rs7305099, rs880054, rs12828016, and rs2051852) were observed in males, and similar finding for rs7305099 was found in females. When comparing the allele distributions, six SNVs (rs4980974, rs7305099, rs880054, rs12828016, rs10774461, and rs7972490) showed significant differences (*p* < 0.05) between the two groups, a borderline founding was also observed for rs2051852 (*p* = 0.05). Furthermore, six SNVs (rs4980974, rs7305099, rs880054, rs12828016, rs2051852, and rs7972490) showed significant differences in allele frequency (*p* < 0.05) in males. In the subgroup of female, statistically significant difference was found for rs7305099 in the allele counts.

### 3.3 Association analyses

Binominal logistic regression analyses were performed under different genetic models (allele comparison, dominant, recessive, homozygote comparison, and additive models) after adjusting for confounding risk variables, including gender, age, BMI, Glu, TG, HDL-C, LDL-C, and UA. The summary of results were listed in Table 2. It showed that the six *WNK1* SNVs (rs7305099, rs880054, rs12828016, rs2051852, rs10774461, and rs7972490) were significantly associated with EH in the allele comparison (rs7305099, *p* < 0.0002, OR = 0.627, 95%CI [0.491–0.801]; rs880054, *p* = 0.008, OR = 0.745, 95%CI [0.600–0.926]; rs12828016, *p* = 0.005, OR = 0.727, 95%CI [0.584–0.907]; rs2051852, *p* = 0.032, OR = 0.773, 95%CI [0.611–0.978]; rs10774461, *p* = 0.002, OR = 0.686, 95%CI [0.538–0.875]; rs7972490, *p* = 0.009, OR = 0.722, 95%CI [0.565–0.922]) (Table 2). As for rs7305099, rs880054, rs12828016, rs10774461, and rs7972490 variants, significant association could also be found in other genetic models (Table 2).

**TABLE 2:** Association of *WNK1* genetic variants with EH under different genetic models after adjustment for confounding factors.

			Overall		Male		Female	
SNV	Models	Contrast	OR (95% CI)^a^	*p* ^a^	OR (95% CI)^a^	*p* ^a^	OR (95% CI)^b^	*p* ^b^
rs11064524	Allele comparison	G vs. T	1.206 (0.973–1.496)	0.088	1.092 (0.822–1.451)	0.542	1.502 (1.064–2.121)	0.021^c^
	Dominant model	GG + GT vs. TT	1.344 (1.004–1.799)	0.047^c^	1.287 (0.874–1.894)	0.202	1.552 (0.973–2.473)	0.065
	Recessive model	GG vs. GT + TT	1.129 (0.710–1.796)	0.607	0.793 (0.424–1.483)	0.467	2.112 (1.013–4.402)	0.046^c^
	Homozygote comparison	GG vs. TT	1.343 (0.820–2.200)	0.241	0.977 (0.500–1.911)	0.947	2.718 (1.210–6.107)	0.015^c^
	Additive models	GG vs. GT vs. TT	1.316 (0.806–2.148)	0.272	0.935 (0.484–1.808)	0.842	2.495 (1.144–5.441)	0.022^c^
rs4980974	Allele comparison	A vs. G	1.188 (0.964–1.464)	0.106	1.315 (0.999–1.732)	0.051	1.029 (0.736–1.439)	0.868
	Dominant model	AA + AG vs. GG	1.336 (0.946–1.886)	0.100	1.322 (0.829–2.110)	0.241	1.488 (0.865–2.562)	0.151
	Recessive model	AA vs. AG + GG	1.192 (0.846–1.681)	0.315	1.547 (1.002–2.386)	0.049^c^	0.669 (0.365–1.225)	0.193
	Homozygote comparison	AA vs. GG	1.356 (0.878–2.096)	0.170	1.701 (0.960–3.015)	0.069	0.927 (0.449–1.917)	0.839
	Additive models	AA vs. AG vs. GG	1.418 (0.930–2.163)	0.105	1.691 (0.976–2.929)	0.061	0.968 (0.473–1.980)	0.929
rs11608756	Allele comparison	A vs. G	0.764 (0.580–1.006)	0.056	0.736 (0.508–1.067)	0.106	0.747 (0.488–1.141)	0.177
	Dominant model	AA + AG vs. GG	0.772 (0.561–1.063)	0.113	0.705 (0.460–1.079)	0.108	0.813 (0.492–1.342)	0.417
	Recessive model	AA vs. AG + GG	0.472 (0.193–1.156)	0.101	0.629 (0.177–2.238)	0.474	0.320 (0.089–1.145)	0.080
	Homozygote comparison	AA vs. GG	0.447 (0.184–1.086)	0.075	0.530 (0.149–1.894)	0.329	0.260 (0.069–0.985)	0.047^c^
	Additive models	AA vs. AG vs. GG	0.444 (0.180–1.093)	0.077	0.570 (0.159–2.038)	0.387	0.311 (0.086–1.129)	0.076
rs7305099	Allele comparison	T vs. G	0.627 (0.491–0.801)	1.873E-4^c^	0.660 (0.480–0.907)	0.010^c^	0.544 (0.363–0.817)	0.003^c^
	Dominant model	TT + GT vs. GG	0.638 (0.471–0.864)	0.004^c^	0.644 (0.433–0.958)	0.030^c^	0.574 (0.347–0.947)	0.030^c^
	Recessive model	TT vs.GT + GG	0.317 (0.162–0.621)	0.001^c^	0.437 (0.194–0.987)	0.047^c^	0.157 (0.043–0.566)	0.005^c^
	Homozygote comparison	TT vs. GG	0.278 (0.140–0.552)	2.584E-4^c^	0.374 (0.162–0.863)	0.021^c^	0.130 (0.034–0.505)	0.003^c^
	Additive models	TT vs. GT vs. GG	0.279 (0.140–0.553)	2.558E-4^c^	0.379 (0.165–0.870)	0.022^c^	0.131 (0.035–0.490)	0.003^c^
rs880054	Allele comparison	G vs. A	0.745 (0.600–0.926)	0.008^c^	0.684 (0.514–0.912)	0.010^c^	0.793 (0.562–1.118)	0.186
	Dominant model	GG + AG vs. AA	0.702 (0.526–0.936)	0.016^c^	0.566 (0.386–0.831)	0.004^c^	0.880 (0.555–1.393)	0.584
	Recessive model	GG vs. AG + AA	0.657 (0.414–1.041)	0.074	0.780 (0.434–1.403)	0.407	0.446 (0.201–0.989)	0.047^c^
	Homozygote comparison	GG vs. AA	0.557 (0.342–0.908)	0.019^c^	0.595 (0.319–1.109)	0.102	0.439 (0.187–1.030)	0.059
	Additive models	GG vs. AG vs. AA	0.568 (0.350–0.922)	0.022^c^	0.594 (0.320–1.101)	0.098	0.448 (0.194–1.034)	0.060
rs12828016	Allele comparison	T vs. G	0.727 (0.584–0.907)	0.005^c^	0.685 (0.513–0.916)	0.011^c^	0.737 (0.517–1.051)	0.092
	Dominant model	TT + GT vs. GG	0.694 (0.521–0.925)	0.013^c^	0.597 (0.408–0.874)	0.008^c^	0.790 (0.498–1.252)	0.316
	Recessive model	TT vs.GT + GG	0.592 (0.359–0.977)	0.040^c^	0.692 (0.370–1.295)	0.249	0.374 (0.152–0.919)	0.032^c^
	Homozygote comparison	TT vs. GG	0.500 (0.295–0.846)	0.010^c^	0.539 (0.277–1.047)	0.068	0.358 (0.139–0.927)	0.034^c^
	Additive models	TT vs. GT vs. GG	0.514 (0.306–0.866)	0.012^c^	0.550 (0.286–1.058)	0.073	0.354 (0.139–0.901)	0.029^c^
rs2051852	Allele comparison	A vs. G	0.773 (0.611–0.978)	0.032^c^	0.716 (0.524–0.980)	0.037^c^	0.790 (0.545–1.145)	0.212
	Dominant model	AA + AG vs. GG	0.755 (0.565–1.009)	0.057	0.630 (0.428–0.927)	0.019^c^	0.849 (0.535–1.345)	0.485
	Recessive model	AA vs. AG + GG	0.630 (0.345–1.149)	0.132	0.838 (0.389–1.806)	0.653	0.401 (0.144–1.120)	0.081
	Homozygote comparison	AA vs. GG	0.566 (0.305–1.047)	0.070	0.695 (0.317–1.521)	0.362	0.395 (0.137–1.138)	0.085
	Additive models	AA vs. AG vs. GG	0.574 (0.311–1.061)	0.076	0.698 (0.319–1.528)	0.368	0.393 (0.138–1.118)	0.080
rs4980973	Allele comparison	A vs. G	1.214 (0.987–1.493)	0.066	1.299 (0.989–1.706)	0.060	1.053 (0.757–1.465)	0.760
	Dominant model	AA + AG vs. GG	1.323 (0.974–1.796)	0.073	1.392 (0.928–2.085)	0.109	1.216 (0.745–1.983)	0.434
	Recessive model	AA vs. AG + GG	1.264 (0.855–1.868)	0.241	1.471 (0.883–2.451)	0.138	0.865 (0.451–1.656)	0.661
	Homozygote comparison	AA vs. GG	1.489 (0.951–2.332)	0.082	1.668 (0.920–3.024)	0.092	1.023 (0.485–2.157)	0.952
	Additive models	AA vs. AG vs. GG	1.464 (0.947–2.265)	0.087	1.714 (0.969–3.030)	0.064	1.008 (0.489–2.080)	0.982
rs10774461	Allele comparison	C vs. A	0.686 (0.538–0.875)	0.002^c^	0.705 (0.513–0.970)	0.032^c^	0.624 (0.420–0.927)	0.020^c^
	Dominant model	CC + AC vs. AA	0.678 (0.505–0.911)	0.010^c^	0.687 (0.466–1.013)	0.058	0.624 (0.386–1.010)	0.055
	Recessive model	CC vs. AC + AA	0.469 (0.245–0.897)	0.022^c^	0.531 (0.232–1.216)	0.134	0.343 (0.113–1.038)	0.058
	Homozygote comparison	CC vs. AA	0.431 (0.224–0.830)	0.012^c^	0.490 (0.211–1.138)	0.097	0.286 (0.087–0.940)	0.039^c^
	Additive models	CC vs. AC vs. AA	0.419 (0.216–0.810)	0.010^c^	0.473 (0.204–1.098)	0.082	0.295 (0.095–0.919)	0.035^c^
rs11611231	Allele comparison	C vs. G	1.195 (0.933–1.531)	0.159	1.030 (0.732–1.449)	0.867	1.495 (1.028–2.173)	0.035^c^
	Dominant model	CC + CG vs. GG	1.234 (0.916–1.662)	0.167	1.012 (0.679–1.509)	0.953	1.746 (1.091–2.792)	0.020^c^
	Recessive model	CC vs. CG + GG	1.247 (0.653–2.385)	0.504	1.203 (0.431–3.361)	0.724	1.277 (0.534–3.052)	0.583
	Homozygote comparison	CC vs. GG	1.349 (0.699–2.606)	0.372	1.205 (0.424–3.420)	0.727	1.636 (0.673–3.975)	0.277
	Additive models	CC vs. CG vs. GG	1.337 (0.693–2.578)	0.387	1.200 (0.426–3.384)	0.730	1.580 (0.647–3.856)	0.315
rs956868	Allele comparison	A vs. C	1.137 (0.865–1.495)	0.357	1.064 (0.729–1.552)	0.749	1.367 (0.903–2.068)	0.140
	Dominant model	AA + AC vs. CC	1.105 (0.805–1.516)	0.537	1.035 (0.675–1.587)	0.874	1.400 (0.849–2.310)	0.188
	Recessive model	AA vs. AC + CC	1.695 (0.719–4.000)	0.228	1.494 (0.409–5.460)	0.544	1.960 (0.586–6.559)	0.275
	Homozygote comparison	AA vs. CC	1.752 (0.742–4.140)	0.201	1.454 (0.406–5.211)	0.565	1.977 (0.562–6.960)	0.288
	Additive models	AA vs. AC vs. CC	1.723 (0.726–4.091)	0.217	1.495 (0.407–5.494)	0.545	2.164 (0.635–7.370)	0.217
rs7972490	Allele comparison	A vs. G	0.722 (0.565–0.922)	0.009^c^	0.706 (0.511–0.976)	0.035^c^	0.685 (0.464–1.011)	0.057
	Dominant model	AA + AG vs. GG	0.710 (0.527–0.956)	0.024^c^	0.629 (0.424–0.933)	0.021^c^	0.736 (0.456–1.189)	0.210
	Recessive model	AA vs. AG + GG	0.534 (0.281–1.013)	0.055	0.790 (0.342–1.823)	0.581	0.316 (0.109–0.915)	0.034^c^
	Homozygote comparison	AA vs. GG	0.478 (0.248–0.922)	0.028^c^	0.650 (0.276–1.532)	0.325	0.299 (0.099–0.900)	0.032^c^
	Additive models	AA vs. AG vs. GG	0.481 (0.250–0.922)	0.028^c^	0.666 (0.284–1.558)	0.348	0.297 (0.101–0.878)	0.028^c^

Abbreviations: CI, confidence interval; OR, odds ratio; SNV, single-nucleotide variant.

^a^ OR, adjusted for gender, age; BMI, glu, HDL-C, LDL-C, TG, and UA.

^b^ OR, adjusted for gender, age; BMI, glu, Hcy; HDL-C, and TG.

^c^ *p* < .05.

In addition, similar results were observed in the subgroup of males for the six SNVs mentioned above under allele comparison (rs7305099, *p* = 0.01, OR = 0.660, 95%CI [0.480–0.907]; rs880054, *p* = 0.01, OR = 0.684, 95%CI [0.514–0.912]; rs12828016, *p* = 0.011, OR = 0.685, 95%CI [0.513–0.916]; rs2051852, *p* = 0.037, OR = 0.716, 95%CI [0.524–0.980]; rs10774461, *p* = 0.032, OR = 0.705, 95%CI [0.513–0.970]; rs7972490, *p* = 0.035, OR = 0.706, 95%CI [0.511–0.976]) (Table 2). In other genetic models, it was found that five SNVs (rs7305099, rs880054, rs12828016, rs2051852, and rs7972490) were significantly associated with EH risk in males (Table 2).

In the female subgroup, we found significant association between the four *WNK1* SNVs (rs11064524, rs7305099, rs10774461, and rs11611231) and EH risk for allele comparison (rs11064524, *p* = 0.021, OR = 1.502, 95%CI [1.064–2.121]; rs7305099, *p* = 0.003, OR = 0.544, 95%CI [0.363–0.817]; rs10774461, *p* = 0.02, OR = 0.624, 95%CI [0.420–0.927]; rs11611231, *p* = 0.035, OR = 1.495, 95%CI [1.028–2.173]) (Table 2). Furthermore, significant associations were observed for the eight *WNK1* SNVs including rs11064524, rs11608756, rs7305099, rs880054, rs12828016, rs10774461, rs11611231, and rs7972490 in females under the other genetic models (Table 2). However, after correcting for multiple testing using Bonferroni [the *p*-value of <0.0008 (0.05/12/5) was considered as significant], the significance remained for the SNV of rs7305099 in three genetic models (allele comparison, *p* < 0.0002, OR = 0.627, 95%CI [0.491–0.801]; homozygote comparison, *p* < 0.0003, OR = 0.278, 95%CI [0.140–0.552]; additive model, *p* < 0.0003, OR = 0.279, 95%CI [0.140–0.553]). Therefore, we concluded that the carriers of rs7305099 T allele had a decreased risk of EH in comparison with the one carrying G allele.

### 3.4 Results of statistical power

Given the available sample size and minor allele frequency, a prospective statistical power of over 80% was achieved for the vast majority of SNV (except for rs4980974, which the value is 0.7355, nearly 80%) for detecting a risk allele with an OR of 1.5. Results for both prospective and *post hoc* statistical power were displayed in Table 2 in the Supplementary Data S1.

### 3.5 Haplotype analyses

**FIGURE 1:**
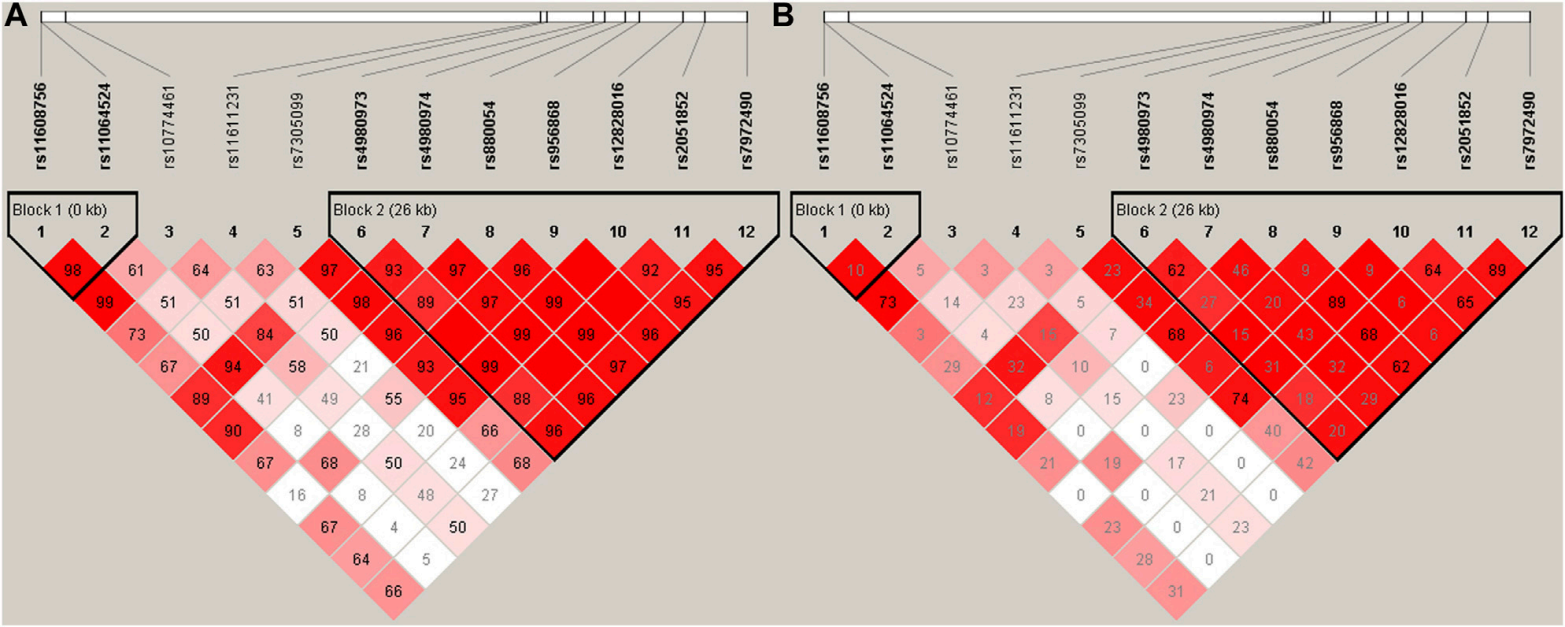
Linkage disequilibrium (LD) blocks defined by the Haploview program based on the solid spine of LD method. **(A)** represents LD results of D’; **(B)** Represents LD results of *r* ^2^.

As shown in Figure 1, two linkage disequilibrium blocks (rs11608756-rs11064524 and rs4980973-rs4980974-rs880054-rs956868-rs12828016-rs2051852-rs7972490) were observed using the Haploview software. Nine haplotypes with frequency greater than 1% were presented in Table 3. The frequency of the haplotype A-A-A-C-G-G-G (rs4980973-rs4980974-rs880054-rs956868-rs12828016-rs2051852-rs7972490) was obviously higher in the hypertensive (42.3%) than in the normotensives (37.8%), but it did not reach statistical significance (*p* = 0.0583). However, after adjustment for the confounding variables (gender, age, BMI, Glu, TG, HDL-C, LDL-C, and UA) with the PLINK software, we found that the haplotype A-A-A-C-G-G-G was significantly associated with increased risk for EH (*p* = 0.043, OR = 1.23) (Table 3).

**TABLE 3:** Haplotype analysis of *WNK1* genetic variants in case and control groups.

									Frequency, %					
Block	SNV1	SNV2	SNV3	SNV4	SNV5	SNV6	SNV7	Haplotype	Case	Control	OR^a^	*P* ^a^	OR^b^	*P* ^b^
1	rs11608756	rs11064524	-	-	-	-	-	G-T	0.486	0.488	0.997	0.976	0.974	0.816
								G-G	0.346	0.316	1.14	0.202	1.11	0.358
								A-T	0.168	0.196	0.828	0.120	0.891	0.407
2	rs4980973	rs4980974	rs880054	rs956868	rs12828016	rs2051852	rs7972490	A-A-A-C-G-G-G	0.423	0.378	1.19	0.058	1.23	0.043^c^
								G-G-G-C-T-A-A	0.211	0.249	0.815	0.059	0.833	0.150
								G-G-A-A-G-G-G	0.173	0.167	1.04	0.768	1.07	0.648
								G-A-A-C-G-G-G	0.108	0.089	1.25	0.161	1.11	0.555
								G-G-G-C-T-G-G	0.057	0.079	0.72	0.079	0.684	0.078
								A-G-G-C-G-A-G	0.011	0.013	0.858	0.724	0.496	0.160

Abbreviations: CI, confidence interval; OR, odds ratio; SNV, single-nucleotide variant.

^a^ ORs, and *p*-values for the haplotype-based association analysis with a specific haplotype compared with the others.

^b^ ORs, and *p*-values for the haplotype-based logistic regression analysis after adjusting for gender, age, BMI, Glu, HDL-C, LDL-C, TG, and UA.

^c^ *p* < .05.

## 4 Discussion

The present study was intended to investigate the relationship between the *WNK1* gene and EH. A total of 12 tagging SNVs of the *WNK1* gene were identified by Haploview software and genotyping was further performed. Binominal logistic regression analyses were also performed to exclude the influences of the confounding factors. Data obtained from this study have shown that the rs7305099 variant in the *WNK1* gene was significantly related to EH risk in the Northern Han Chinese population after Bonferroni correction for multiple testing, and T allele was a protective factor for hypertension. In the two haplotype blocks, the rs4980973-rs4980974-rs880054-rs956868-rs12828016-rs2051852-rs7972490 A-A-A-C-G-G-G haplotype might be a risk factor for EH. It is worth noting that hypertensive subjects taking antihypertensive medication were included in the current study, accounting for 78.57% of hypertensive patients. Accordingly, the mean SBP value of all patients was quite low. However, 21.43% of individuals did not take medication or take antihypertensive drugs irregularly, this also reminded us to further exhort patients to take medicine as directed.

EH is a multifactorial disease, involving the interaction of multiple genes and environmental factors (Wang et al., 2020). Ample evidence supports a crucial role for the kidney in BP regulation and pathogenesis of EH (Rossier et al., 2013; Franceschini et al., 2014). In particular, several rare Mendelian forms of hypertension are proved to be due to genetic mutations involved in sodium and water reabsorption (Lifton et al., 2001; Delles et al., 2010). WNK1 is a serine-threonine kinase, it can regulate various ion channels, which participate in the sodium and chloride transport in the kidney (Náray-F et al., 2004; Cope et al., 2006). NCC is a critical sodium reabsorption channel located in the DCT, and it is a key regulator resulting in volume expansion and hypertension when it is hyperactive (Simon et al., 1996; Subramanya et al., 2014). The reabsorption of NCC must be switched on with direct phosphorylation by Ste20-like proline-alanine rich kinase (SPAK) and oxidative stress responsive kinase 1 (OSR1), phosphorylated and activated by a family of proteins called WNKs (Richardson et al., 2008). *WNK1* mutations cause increased expression of L-WNK1 in the distal tubule (Brown et al., 2021). Therefore, the WNK-SPAK/OSR1-NCC pathway is overactivated, and NaCl reabsorption is increased, leading to hypertension (Brown et al., 2021).

A number of potential candidate genes for BP regulation and EH have been identified through studies of rare Mendelian forms of hypertension. Previous studies also found the possible relationship between *WNK1* SNVs and EH risk, and several SNVs have been included in the current study. In Kokubo et al. (2004) selected seven *WNK1* SNVs (rs956868, rs3858703, and rs2286007 et al.) and investigated the association between these SNVs and EH risk in the Japanese general population. The results suggest no association of *WNK1* with hypertension was observed (Kokubo et al., 2004). Similarly, a family-based association study by Newhouse et al. (2005) demonstrated no relationship with hypertension was found for the *WNK1* rs956868 variant in white European populations. However, Han and Hui (2008) reported the increased risk of EH was found to be associated with rs956868 of *WNK1* in the Han Chinese population after adjustment for conventional risk factors by logistic regression analysis (dominant model, AA + AC vs. CC: *p* = 0.007, OR = 1.39, 95%CI [1.10–1.77]). Unfortunately, our present study failed to find a positive connection between *WNK1* rs956868 variant and HT risk in the Northern Han Chinese. Rs956868 is located on the exon 13 of the *WNK1* gene, and its association with the genetic susceptibility to hypertension has been reported in various ethnic groups. The following factors may explain the observed negative result for rs956868 of *WNK1* and EH. First, lack of association may result from ethnic differences. Different races may have different genetic backgrounds and heterogeneity. In the above studies, the frequency of A allele for rs956868 was 0.134 in the Japanese population, and 0.177 in the British white population. In addition, the data reported by Han et al. was 0.234 in the Chinese Han population, whereas the frequency of A allele in our current study was 0.174 which is similar to that reported in the British white population. Second, we cannot rule out the possibility that the results have limitations due to sampling bias. Furthermore, it is worth mentioning that another possibility is a “true” mutation, as yet unidentified, exists in the linkage disequilibrium with rs956868. It is expected that further studies with large samples will be conducted in the future.

Rs880054 is another SNV of *WNK1*, and it is located in intron 10. Tobin et al. (2005) reported that the rs880054 contributes to BP variation in a population-based sample of 996 subjects from 250 white European families. However, a Chinese study on Tibetan population has shown that both rs880054 and rs4980973 mutations were not associated with EH susceptibility (Shi et al., 2018). Consistently, no significant connections between *WNK1* genetic variants (rs880054 and rs4980973) and EH were found in our study. As reported, the minimum allele frequencies of rs880054 and rs4980973 were 0.323 and 0.418 respectively in the current study population. In the Tibetan ethnic group of China, the data were 0.33 and 0.42 respectively (Shi et al., 2018), which were in accord with the Northern Han Chinese. However, the minimum allele frequency of rs880054 in white European subjects was 43.8%, according to Tobin’s Report (Tobin et al., 2005). As we all know, EH is the result of the joint influence of both genes and environmental factors (Zhang et al., 2022). Genetic background, growth environment and dietary habits et al. are all the root causes of ethnic heterogeneity, the influence of confounding factors should be more rigorously controlled to increase the accuracy of the results (Shi et al., 2021). In view of this, the findings need to be validated in other populations.

Notably, rs7305099, located in the eighth intron of the *WNK1* gene, was found to be significantly correlated with EH in our study. It is not surprising that most tag SNVs detected by Haplotype software locate in the introns of gene (Sakharkar et al., 2004). Although introns can not be translated into amino acids directly, their key role in gene expression regulation cannot be ignored (Le Hir et al., 2003; Jo and Choi, 2015). Mutation of the rs7305099 locus could elevate the expression of *WNK1*, and may therefore be involved in the pathogenesis of EH. Alternatively, tag SNV located in intron maybe just a site which was in close linkage disequilibrium (LD) with the other functional genetic variants (Dong et al., 2017; Grossi et al., 2018). With the consideration of these findings, the potential function of the rs7305099 variant was further predicted by using HaploReg (version 4.0) (http://pubs.broadinstitute.org/mammals/haploreg/haploreg_v4.php). It was found that there were 9 SNVs in LD with rs7305099. These SNVs are reported to be associated with enhancer histone marks, DNAse, proteins bound, motifs changed and selected expression Quantitative Trait Loci (eQTL) hits, many of which are involved in the regulation of blood pressure (Manunta et al., 2008; Liu et al., 2013). Of course, the functional role of rs7305099 in EH should be verified in future studies.

Since the interaction of multiple SNVs on the biological phenotype, haplotype analysis was further performed in the present study, which may be more powerful to identify genetic variants of complex diseases, such as EH. The present haplotype analysis results showed that the haplotype A-A-A-C-G-G-G (rs4980973-rs4980974-rs880054-rs956868-rs12828016-rs2051852-rs7972490) was significantly associated with increased risk for EH.

Our research team has been committed to the study of SNVs for EH. According to a previous study by Li et al. (2019) the prediction models to recognize individuals with high risk for EH were presented, and rs7305099 was identified to be a genetic factor for EH prediction, which was consistent with our present findings. Although it is undeniable that some hypertensive subjects were from the Anzhen community in our study, which overlapped with Li et al.’s, they were two distinct studies with different designs. Firstly, our research was designed in a community based trial, and subjects were from the community of Anzhen, Liuliqiao and Guozhuang in Beijing. While the subjects in Li et al.’s study were hospital based patients, who were screened in clinic and physical examination center at Anzhen hospital. In general, community or population-based sampling strategy is more representative and brings less bias compared to the hospital-based study. Secondly, the purposes and research perspectives of the two studies were different. Li et al.’s study aimed to develop prediction models to recognize individuals with high risk for EH or BP. Thus many variables for genes, SNVs, and other factors were included. By contrast, our present study focused on genetic patterns of *WNK1* and feature of haplotypes between hypertensives and controls. To some extent, the present research can be seen as a validation of Li et al.’s study in community-based design.

Although both inner authenticity and the representativeness of the sampling population were considered in the current study, several limitations still existed. First, it is worth noting that EH is regarded as a complex polygenic disease responsive to environmental factors. A SNV or gene likely has weak effects on the individual’s phenotype, because both genetic and environmental factors have been implicated in the occurrence and development of EH, as well as their interactions. This was the main limitation of the study. Second, the present study covered most of the common *WNK1* variants, but, unavoidably, some regulatory regions and functional domains might be missed, especially other functional SNVs with lower frequency that are still worthy of study. Third, although the subjects were adequately matched for age and gender between groups, some participants in control group might inevitably develop EH later in the life. Fourth, the study was performed in only one region (Beijing), which may cause some geographic bias. Fifth, considering low statistical power for several SNVs in our study, caution should be exercised when extrapolating the insignificant association between the SNV for *WNK1* and hypertension. Therefore, in future studies, evaluation of potential interactions such as gene-gene, gene-environment is required in countrywide large samples to the pathological mechanism behind the correlation. Results produced can only demonstrate correlations rather than causality.

In conclusion, the rs7305099 T allele of *WNK1* gene conferred to a significantly decreased risk for EH in the Northern Han Chinese population in Beijing, and individuals with haplotype A-A-A-C-G-G-G (rs4980973-rs4980974-rs880054-rs956868-rs12828016-rs2051852-rs7972490) are more susceptible to developing EH. These could provide evidences to the risk assessment, early prevention and individualized therapy of EH to some extent. Further functional studies are needed to confirm above discovery, and should be testified in different populations.

## Data Availability

The raw data supporting the conclusion of this article will be made available by the authors, without undue reservation.
