# Supplementary figures and images for: Association study of WNK1 genetic variants and essential hypertension risk in the Northern Han Chinese in Beijing
Source: Front Genet. 2023 Sep 15;14:1234536. doi: 10.3389/fgene.2023.1234536 (PMC10541150; doi:10.3389/fgene.2023.1234536)

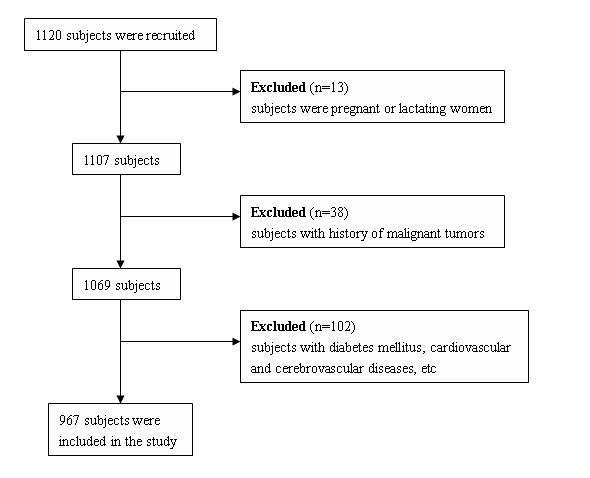

Supplement: Supplementary file 1 [file Image1.JPEG]
