# Supplementary material for: Association study of WNK1 genetic variants and essential hypertension risk in the Northern Han Chinese in Beijing
Source: Front Genet. 2023 Sep 15;14:1234536. doi: 10.3389/fgene.2023.1234536 (PMC10541150; doi:10.3389/fgene.2023.1234536)
Supplement: Supplementary file 2 [file Table1.doc]

**Supplement-Table 1** Distribution of allelic and genotypic frequencies of *WNK1* SNVs and HWE information

|  | | | | | | | | **HWE** | | **GSR**  **(%)** |
| --- | --- | --- | --- | --- | --- | --- | --- | --- | --- | --- |
| **SNV** | **Genotype (****frequency, %)** | | | ***P*** | **Allele (frequency, %)** | | ***P*** | **χ^2^** | ***P*** |
| **rs11064524** | **GG** | **GT** | **TT** |  | **G** | **T** |  |  |  | 0.994 |
| **Overall** |  |  |  | 0.251 |  |  | 0.151 |  |  |  |
| **Case** | 52(11.1) | 223(47.3) | 196(41.6) |  | 327(34.7) | 615(65.3) |  |  |  |  |
| **Control** | 50(10.2) | 210(42.9) | 230(46.9) |  | 310(31.6) | 670(68.4) |  | 0.041 | 0.839 |  |
| **Male** |  |  |  | 0.264 |  |  | 0.404 |  |  |  |
| **Case** | 26(9.5) | 136(49.8) | 111(40.7) |  | 188(34.4) | 358(65.6) |  |  |  |  |
| **Control** | 29(10.6) | 117(42.9) | 127(46.5) |  | 175(32.1) | 371(67.9) |  | 0.070 | 0.791 |  |
| **Female** |  |  |  | 0.452 |  |  | 0.222 |  |  |  |
| **Case** | 26(13.1) | 87(44.0) | 85(42.9) |  | 139(35.1) | 257(64.9) |  |  |  |  |
| **Control** | 21(9.7) | 93(42.9) | 103(47.4) |  | 135(31.1) | 299(68.9) |  | 1.200E-6 | 0.999 |  |
| **rs4980974** | **AA** | **AG** | **GG** |  | **A** | **G** |  |  |  | 0.945 |
| **Overall** |  |  |  | 0.075 |  |  | 0.027^*^ |  |  |  |
| **Case** | 125(28.2) | 221(49.9) | 97(21.9) |  | 471(53.2) | 415(46.8) |  |  |  |  |
| **Control** | 114(24.1) | 227(47.9) | 133(28.0) |  | 455(48.0) | 493(52.0) |  | 0.783 | 0.376 |  |
| **Male** |  |  |  | 0.054 |  |  | 0.024^*^ |  |  |  |
| **Case** | 85(33.1) | 118(45.9) | 54(21.0) |  | 288(56.0) | 226(44.0) |  |  |  |  |
| **Control** | 62(23.7) | 133(50.8) | 67(25.5) |  | 257(49.0) | 267(51.0) |  | 0.064 | 0.800 |  |
| **Female** |  |  |  | 0.076 |  |  | 0.482 |  |  |  |
| **Case** | 40(21.5) | 103(55.4) | 43(23.1) |  | 183(49.2) | 189(50.8) |  |  |  |  |
| **Control** | 52(24.5) | 94(44.4) | 66(31.1) |  | 198(46.7) | 226(53.3) |  | 2.534 | 0.111 |  |
| **rs11608756** | **AA** | **AG** | **GG** |  | **A** | **G** |  |  |  | 0.933 |
| **Overall** |  |  |  | 0.189 |  |  | 0.121 |  |  |  |
| **Case** | 11(2.5) | 127(28.6) | 306(68.9) |  | 149(16.8) | 739(83.2) |  |  |  |  |
| **Control** | 21(4.5) | 139(30.1) | 302(65.4) |  | 181(19.6) | 743(80.4) |  | 0.934 | 0.334 |  |
| **Male** |  |  |  | 0.295 |  |  | 0.127 |  |  |  |
| **Case** | 6(2.3) | 67(26.1) | 184(71.6) |  | 79(15.4) | 435(84.6) |  |  |  |  |
| **Control** | 8(3.2) | 80(31.6) | 165(65.2) |  | 96(19.0) | 410(81.0) |  | 0.205 | 0.651 |  |
| **Female** |  |  |  | 0.200 |  |  | 0.567 |  |  |  |
| **Case** | 5(2.7) | 60(32.1) | 122(65.2) |  | 70(18.7) | 304(81.3) |  |  |  |  |
| **Control** | 13(6.2) | 59(28.2) | 137(65.6) |  | 85(20.3) | 333(79.7) |  | 3.462 | 0.063 |  |
| **rs7305099** | **TT** | **GT** | **GG** |  | **T** | **G** |  |  |  | 0.924 |
| **Overall** |  |  |  | 0.001^*^ |  |  | <0.001^*^ |  |  |  |
| **Case** | 17(3.9) | 154(35.7) | 261(60.4) |  | 188(21.8) | 676(78.2) |  |  |  |  |
| **Control** | 43(9.2) | 186(39.8) | 238(51.0) |  | 272(29.1) | 662(70.9) |  | 0.579 | 0.447 |  |
| **Male** |  |  |  | 0.011^*^ |  |  | 0.003^*^ |  |  |  |
| **Case** | 11(4.4) | 87(34.5) | 154(61.1) |  | 109(21.6) | 395(78.4) |  |  |  |  |
| **Control** | 23(8.9) | 108(41.9) | 127(49.2) |  | 154(29.8) | 362(70.2) |  | 3.320E-5 | 0.995 |  |
| **Female** |  |  |  | 0.042^*^ |  |  | 0.044^*^ |  |  |  |
| **Case** | 6(3.3) | 67(37.2) | 107(59.5) |  | 79(21.9) | 281(78.1) |  |  |  |  |
| **Control** | 20(9.6) | 78(37.3) | 111(53.1) |  | 118(28.2) | 300(71.8) |  | 1.304 | 0.254 |  |
| **rs880054** | **GG** | **AG** | **AA** |  | **G** | **A** |  |  |  | 0.991 |
| **Overall** |  |  |  | 0.029^*^ |  |  | 0.007^*^ |  |  |  |
| **Case** | 42(8.8) | 196(41.3) | 237(49.9) |  | 280(29.5) | 670(70.5) |  |  |  |  |
| **Control** | 62(12.8) | 216(44.7) | 205(42.5) |  | 340(35.2) | 626(64.8) |  | 0.187 | 0.666 |  |
| **Male** |  |  |  | 0.004^*^ |  |  | 0.002^*^ |  |  |  |
| **Case** | 26(9.5) | 100(36.5) | 148(54.0) |  | 152(27.7) | 396(72.3) |  |  |  |  |
| **Control** | 35(13.0) | 127(47.2) | 107(39.8) |  | 197(36.6) | 341(63.4) |  | 0.079 | 0.779 |  |
| **Female** |  |  |  | 0.211 |  |  | 0.630 |  |  |  |
| **Case** | 16(8.0) | 96(47.7) | 89(44.3) |  | 128(31.8) | 274(68.2) |  |  |  |  |
| **Control** | 27(12.6) | 89(41.6) | 98(45.8) |  | 143(33.4) | 285(66.6) |  | 0.914 | 0.339 |  |
| **rs12828016** | **TT** | **GT** | **GG** |  | **T** | **G** |  |  |  | 0.998 |
| **Overall** |  |  |  | 0.022^*^ |  |  | 0.006^*^ |  |  |  |
| **Case** | 35(7.4) | 191(40.3) | 248(52.3) |  | 261(27.5) | 687(72.5) |  |  |  |  |
| **Control** | 55(11.2) | 217(44.2) | 219(44.6) |  | 327(33.3) | 655(66.7) |  | 0.013 | 0.910 |  |
| **Male** |  |  |  | 0.005^*^ |  |  | 0.002^*^ |  |  |  |
| **Case** | 22(8.1) | 100(36.6) | 151(55.3) |  | 144(26.4) | 402(73.6) |  |  |  |  |
| **Control** | 32(11.7) | 128(46.7) | 114(41.6) |  | 192(35.0) | 356(65.0) |  | 0.188 | 0.664 |  |
| **Female** |  |  |  | 0.285 |  |  | 0.529 |  |  |  |
| **Case** | 13(6.5) | 91(45.3) | 97(48.2) |  | 117(29.1) | 285(70.9) |  |  |  |  |
| **Control** | 23(10.6) | 89(41.0) | 105(48.4) |  | 135(31.1) | 299(68.9) |  | 0.403 | 0.526 |  |
| **rs2051852** | **AA** | **AG** | **GG** |  | **A** | **G** |  |  |  | 0.997 |
| **Overall** |  |  |  | 0.131 |  |  | 0.050^*^ |  |  |  |
| **Case** | 23(4.9) | 169(35.7) | 281(59.4) |  | 215(22.7) | 731(77.3) |  |  |  |  |
| **Control** | 36(7.3) | 189(38.5) | 266(54.2) |  | 261(26.6) | 721(73.4) |  | 0.093 | 0.761 |  |
| **Male** |  |  |  | 0.046^*^ |  |  | 0.018^*^ |  |  |  |
| **Case** | 14(5.1) | 87(31.9) | 172(63.0) |  | 115(21.1) | 431(78.9) |  |  |  |  |
| **Control** | 19(6.9) | 111(40.5) | 144(52.6) |  | 149(27.2) | 399(72.8) |  | 0.147 | 0.701 |  |
| **Female** |  |  |  | 0.272 |  |  | 0.789 |  |  |  |
| **Case** | 9(4.5) | 82(41.0) | 109(54.5) |  | 100(25.0) | 300(75.0) |  |  |  |  |
| **Control** | 17(7.8) | 78(36.0) | 122(56.2) |  | 112(25.8) | 322(74.2) |  | 0.816 | 0.366 |  |
| **rs4980973** | **AA** | **AG** | **GG** |  | **A** | **G** |  |  |  | 0.991 |
| **Overall** |  |  |  | 0.193 |  |  | 0.073 |  |  |  |
| **Case** | 89(18.9) | 236(50.0) | 147(31.1) |  | 414(43.9) | 530(56.1) |  |  |  |  |
| **Control** | 77(15.8) | 233(48.0) | 176(36.2) |  | 387(39.8) | 585(60.2) |  | 6.220E-5 | 0.994 |  |
| **Male** |  |  |  | 0.153 |  |  | 0.062 |  |  |  |
| **Case** | 56(20.5) | 135(49.5) | 82(30.0) |  | 247(45.2) | 299(54.8) |  |  |  |  |
| **Control** | 40(14.8) | 134(49.6) | 96(35.6) |  | 214(40.0) | 326(60.0) |  | 0.374 | 0.541 |  |
| **Female** |  |  |  | 0.575 |  |  | 0.575 |  |  |  |
| **Case** | 33(16.6) | 101(50.7) | 65(32.7) |  | 167(42.0) | 231(58.0) |  |  |  |  |
| **Control** | 37(17.1) | 99(45.8) | 80(37.1) |  | 173(40.0) | 259(60.0) |  | 0.447 | 0.504 |  |
| **rs10774461** | **CC** | **AC** | **AA** |  | **C** | **A** |  |  |  | 0.997 |
| **Overall** |  |  |  | 0.115 |  |  | 0.041^*^ |  |  |  |
| **Case** | 21(4.4) | 156(33.0) | 296(62.6) |  | 198(20.9) | 748(79.1) |  |  |  |  |
| **Control** | 34(7.0) | 176(35.8) | 281(57.2) |  | 244(24.8) | 738(75.2) |  | 0.794 | 0.373 |  |
| **Male** |  |  |  | 0.272 |  |  | 0.111 |  |  |  |
| **Case** | 13(4.8) | 87(32.0) | 172(63.2) |  | 113(20.8) | 431(79.2) |  |  |  |  |
| **Control** | 17(6.2) | 102(37.2) | 155(56.6) |  | 136(24.8) | 412(75.2) |  | 0.002 | 0.968 |  |
| **Female** |  |  |  | 0.244 |  |  | 0.200 |  |  |  |
| **Case** | 8(4.0) | 69(34.3) | 124(61.7) |  | 85(21.1) | 317(78.9) |  |  |  |  |
| **Control** | 17(7.8) | 74(34.1) | 126(58.1) |  | 108(24.9) | 326(75.1) |  | 1.674 | 0.196 |  |
| **rs11611231** | **CC** | **CG** | **GG** |  | **C** | **G** |  |  |  | 0.988 |
| **Overall** |  |  |  | 0.402 |  |  | 0.178 |  |  |  |
| **Case** | 25(5.4) | 161(34.6) | 279(60.0) |  | 211(22.7) | 719(77.3) |  |  |  |  |
| **Control** | 22(4.5) | 154(31.4) | 315(64.1) |  | 198(20.2) | 784(79.8) |  | 0.327 | 0.568 |  |
| **Male** |  |  |  | 0.996 |  |  | 0.933 |  |  |  |
| **Case** | 10(3.8) | 83(31.2) | 173(65.0) |  | 103(19.4) | 429(80.6) |  |  |  |  |
| **Control** | 10(3.6) | 85(31.1) | 179(65.3) |  | 105(19.2) | 443(80.8) |  | 0.535E-3 | 0.982 |  |
| **Female** |  |  |  | 0.147 |  |  | 0.055 |  |  |  |
| **Case** | 15(7.5) | 78(39.2) | 106(53.3) |  | 108(27.1) | 290(72.9) |  |  |  |  |
| **Control** | 12(5.5) | 69(31.8) | 136(62.7) |  | 93(21.4) | 341(78.6) |  | 0.674 | 0.412 |  |
| **rs956868** | **AA** | **AC** | **CC** |  | **A** | **C** |  |  |  | 0.966 |
| **Overall** |  |  |  | 0.573 |  |  | 0.716 |  |  |  |
| **Case** | 15(3.4) | 128(28.7) | 303(67.9) |  | 158(17.7) | 734(82.3) |  |  |  |  |
| **Control** | 11(2.2) | 145(29.7) | 333(68.1) |  | 167(17.1) | 811(82.9) |  | 1.083 | 0.298 |  |
| **Male** |  |  |  | 0.771 |  |  | 0.569 |  |  |  |
| **Case** | 7(2.7) | 72(27.6) | 182(69.7) |  | 86(16.5) | 436(83.5) |  |  |  |  |
| **Control** | 5(1.8) | 73(26.7) | 195(71.5) |  | 83(15.2) | 463(84.8) |  | 0.377 | 0.539 |  |
| **Female** |  |  |  | 0.603 |  |  | 0.996 |  |  |  |
| **Case** | 8(4.3) | 56(30.3) | 121(65.4) |  | 72(19.5) | 298(80.5) |  |  |  |  |
| **Control** | 6(2.8) | 72(33.3) | 138(63.9) |  | 84(19.4) | 348(80.6) |  | 0.886 | 0.347 |  |
| **rs7972490** | **AA** | **AG** | **GG** |  | **A** | **G** |  |  |  | 0.968 |
| **Overall** |  |  |  | 0.047^*^ |  |  | 0.021^*^ |  |  |  |
| **Case** | 19(4.2) | 155(34.5) | 276(61.3) |  | 193(21.4) | 707(78.6) |  |  |  |  |
| **Control** | 37(7.6) | 179(36.8) | 271(55.6) |  | 253(26.0) | 721(74.0) |  | 0.952 | 0.329 |  |
| **Male** |  |  |  | 0.068 |  |  | 0.022^*^ |  |  |  |
| **Case** | 11(4.2) | 82(31.3) | 169(64.5) |  | 104(19.8) | 420(80.2) |  |  |  |  |
| **Control** | 17(6.3) | 105(38.9) | 148(54.8) |  | 139(25.7) | 401(74.3) |  | 0.080 | 0.777 |  |
| **Female** |  |  |  | 0.122 |  |  | 0.395 |  |  |  |
| **Case** | 8(4.3) | 73(38.8) | 107(56.9) |  | 89(23.7) | 287(76.3) |  |  |  |  |
| **Control** | 20(9.2) | 74(34.1) | 123(56.7) |  | 114(26.3) | 320(73.7) |  | 3.105 | 0.078 |  |

Abbreviations: SNV, single-nucleotide variant; HWE, Hardy–Weinberg equilibrium; GSR, genotyping success rate.

*p < .05.
