# Supplementary material for: Association study of WNK1 genetic variants and essential hypertension risk in the Northern Han Chinese in Beijing
Source: Front Genet. 2023 Sep 15;14:1234536. doi: 10.3389/fgene.2023.1234536 (PMC10541150; doi:10.3389/fgene.2023.1234536)
Supplement: Supplementary file 3 [file Table2.DOC]

**Supplement-Table 2 Results for prospective and post-hoc statistical power**

| **SNV** | **rs11064524** | **rs4980974** | **rs11608756** | **rs7305099** | **rs880054** | **rs12828016** | **rs2051852** | **rs4980973** | **rs10774461** | **rs11611231** | **rs956868** | **rs7972490** |
| --- | --- | --- | --- | --- | --- | --- | --- | --- | --- | --- | --- | --- |
| **prospective**  **SP** | 0.871 | 0.736 | 0.829 | 0.856 | 0.873 | 0.879 | 0.879 | 0.827 | 0.875 | 0.867 | 0.835 | 0.868 |
| **post-hoc**  **SP** | 0.619 | 0.464 | 0.456 | 0.918 | 0.774 | 0.806 | 0.584 | 0.524 | 0.845 | 0.355 | 0.11 | 0.737 |

Abbreviations: SNV, single-nucleotide variant; SP, statistical power.
